# Surgical Essays, the Result of Clinical Observations Made at Guy's Hospital

**Published:** 1834-01-01

**Authors:** 


### Surgical Essays, the Result of Clinical Observations made at Guy's Hospital. By *Bransby B. Cooper*, F.R.S. &c. Large 8vo. pp. 281. London, 1833.

The object of this publication is, to use the words of its zealous author, to give to the world a collection of cases occurring in hospital practice, systematically arranged, with the views that immediately influenced the method of treatment. Mr. Cooper compares a report thus conducted to a series of clinical lectures, or rather to the preparations in a well-arranged museum. He remarks, and remarks truly, that the benefits conferred by the establishment of hospitals are double—one, ostensible and great, to the afflicted poor—the other, less ostensible to laymen, but almost equally important, to the youth of the profession.

We hail the appearance of a work professing principles like these with a feeling of great satisfaction. It is but the beginning of the end, a prelude to other things. We have urged on hospital surgeons and physicians, the duty and the necessity of making the facts which they observe subservient to public professional utility. Till this work came, our call was answered only from the provinces, and the lethargy of aristocratical reserve was not interrupted in the Capital. But the signs of the times make an earlier impression on some men than on others, and the striking manifestations of public feeling that have recently been witnessed, must induce those who rank as the foremost in the profession to bestir themselves, and answer its just expectations.

Hospital appointments must soon be considered as real trusts, not only to the governors and patients of the institutions, but also to science and the medical public. They must not be longer looked on as preserves, where a favoured race may be fattened and fed with little exertion or merit of their own. Our professional youth have a right to ask for cheaper opportunities of acquiring knowledge—the members of our colleges should be free of the practice of every hospital—and the medical officers should deem themselves bound to communicate clinical reports, in the fullest and most eligible manner. We have little doubt that few years will elapse, before the impatient spirit of the day will demand and obtain such concessions as these.

We have spoken so often and so long on the subject of clinical reports, that further observations would be merely repetition. We know not if our efforts to encourage the surgeons and physicians of hospitals to collect and to diffuse them, have been productive of desirable consequences. But our voice has been earnest, if it has not been efficient, and vanity might tempt us to express the suspicion, that the zealous have been stimulated and encouraged by our praise—the indolent shamed by the severity of our censure.

To ensure a reasonable quantum of success, an undertaking commenced with ardour must be carried into execution with discretion. It is not enough that the spirit be willing—the flesh also must be strong. When we urge the publication of clinical reports, we are far from wishing to entrap the reporters into a ruinous or needless expense. It is flattering to our pride to convey our actions or opinions to the world, in the manner most adapted to our consciousness of merit. But the world is more niggard of its praise than of its censure—of its practical and real support than of its praise. We are tempted to indulge in this strain of remark, from observing, with more of misgiving than surprise, that the gentlemen who have lately published clinical reports have embodied them in regular—indeed in voluminous works. We think that, if pecuniary expense is an object, the plan is attended with serious objections. The quantity of volumes that issue from the press preclude the extensive purchase of many, and those which are devoted to the mere collection of insulated, or comparatively insulated, facts, are beyond the desire and the reach of buyers. Even should the authors be so disinterested, and we do not say that they are not, as to disregard their personal loss, and merge their purses and their minds in a keen aspiration for general advantage, we would hint that books which are little bought are very frequently little read. The diffusion of their views and of their names is necessary, we imagine, to their extensive reputation, and thus we may perceive, that the mingled operation of poverty and bad taste on the part of the reader, wounds the writer in two very sensible places—his pocket and his fame. If the pointed, and perhaps unpleasant, question were put to Dr. Macfarlane, Mr. Fleming, or Mr. Fletcher — "Have your clinical reports paid the cost of their paper?" we fear that their reply would be, "No, they have not." And yet these reports are possessed of great value to all who rightly appreciate facts, and are actuated by the spirit of inductive philosophy.

Although it is easy to point out the error which persons of ability and excellent intentions have committed, it is not easy to detect the remedy. It may be said that the periodical journals present an obvious and appropriate medium for the publication of clinical facts. But the only journals which possess a circulation are those of a quarterly and weekly form. However extended may be the circulation of the latter, their rapid succession and numerous phases contribute greatly to distract attention, and to give an ephemeral character to their contents. Their bulk is also ill adapted to the insertion of lengthened papers. There are equal objections to the selection of journals of a quarterly description, but the fear of appearing censorious or invidious prevents particular allusion to their nature.

Perhaps a volume, resembling the Transactions of the Medico-Chirurgical Society, would form the best vehicle of clinical reports. The expense might be defrayed in one of two manners—the Colleges of Surgeons and Physicians, reformed and adapted to the wants of the profession, or their single successor, if a single successor should appear, might superintend the publication of the work, and devote the collegiate funds to its expenses. Or the hospital surgeons and physicians themselves, comprising those of the provinces and capital, might constitute an association for this express purpose. By this step they would shew the spirit which would animate them, acquire a title to the grateful consideration of their brethren, and remove a portion of that odium, probably unmerited, which industrious efforts on the part of their opponents have successfully excited against them.

We commenced with a promise that we would not digress, but we fear that the promise has been broken. Insensibly led away by our zeal for the promotion of useful professional knowledge, we have ventured to offer exhortation and advice, where our business was strictly review and analysis.

The work before us consists of what may be considered as five separate and distinct essays. The first is devoted to the Physiology of the Growth and Reparation of Bone—the second to Fractures in general—the third to Diseases of Joints—the fourth to Dislocations—and the fifth to Wounds and Injuries of the Abdomen. We select the latter for our present notice, and our choice is determined by its brevity rather than its worth. It would not be possible, were it even advantageous, to offer an analytical account of a clinical report of nearly three hundred pages. But the nature of the work admits of its dismemberment, and the fragments can be offered at convenient opportunities in convenient places. We shall give a short account of the Essay on Wounds of the Abdomen here, and the most important of the various facts profusely scattered through the pages of the volume, will be found at various times in our clinical department.

Wounds of the abdomen are considered by Mr. Cooper, as they have been considered by others, under different heads. The first comprises simple contusion of the abdominal parietes—the second, wounds of the parietes—the third, wounds of the parietes, with protrusion of the viscera—the fourth, wounds of the parietes and viscera—the fifth, laceration of the viscera, without solution of continuity of the parietes.

Of mere contusion of the abdominal parietes little need be said. When the patient is admitted in the state of collapse, there are few, and perhaps there may be no indications of injury of the viscera, or parts within. But Mr. Cooper justly remarks that, if there be lesion, the subsequent re-action tends to display it. The object of the surgeon is to prevent inflammation, by the use of appropriate and prompt depletion so soon as re-action begins.

We need not advert to the treatment recommended by our author for wounds of the parietes, without protrusion of the contents of the abdomen. They are those which are commonly adopted. If there is reason to suspect that the intestine has been injured, purgative medicines should be carefully avoided, at least in the first instance. Our author adverts to the treatment of intestine, when protruded through a wound. His considerations have been so much anticipated by most of the writers upon hernia, that we need not detail them here.

The following case displays the supervention of peritonitis on protrusion of intestine, although the protruded portion itself was not, in any especial degree, the seat of inflammation.

*Case.* A man, while stealing lead from a brewery, was precipitated from its top. He was taken to Guy's Hospital. One thigh was broken, the left shoulder dislocated, and an old scrotal hernia was torn open, a considerable quantity of intestine having protruded, and remained exposed for nearly an hour. The intestine was immediately returned into the cavity of the abdomen, and the edges of the wound brought together by the uninterrupted suture; the fractured thigh was placed in splints, and the dislocated shoulder reduced, which was accomplished with much more than usual facility, in consequence of the state of collapse of the patient from his abdominal injury. His pulse being feeble, the surface of his body cold, and his respiration difficult, julep. ammon. was administered, and bottles of hot water applied to his feet, for the purpose of producing re-action, which was no sooner effected than pain in the abdomen came on, for which leeches were applied, and calomel with opium given, for the purpose of allaying his pain; all the symptoms, however, rapidly increased in urgency, and in fifteen hours after his admission he died.

Examination of the body disclosed severe peritoneal inflammation. The protruded intestine had not been ruptured, nor could it be distinguished from the remainder of the gut, excepting by its thickening, probably the result of its frequent descent into the hernial sac. The diaphragm was ruptured, and a considerable portion of the stomach had passed into the chest, a circumstance of which the symptoms had occasioned no suspicion during life.

"Several cases are on record of viscera having protruded through incised wounds of the abdomen, in which they have been returned into their natural cavity, and without any alarming symptom supervening, where a strict antiphlogistic discipline had been employed for the purpose of preventing inflammation. I have heard my colleague, Mr. Morgan, relate a case, in which a boy at Tottenham received a wound in the abdomen, through which a large quantity of intestine protruded; he placed the viscera in his pinbefore, and walked a considerable distance to a surgeon who freed the bowels from a quantity of dust which adhered to them, returned them into the cavity of the abdomen, and sewed up the wound by the uninterrupted suture; by this judicious treatment the patient was restored to health." 264.

Mr. Cooper alludes to the practice of Mr. Key in cases of strangulated hernia, a practice of which full notice has been taken in the present number of this journal. He appears to be an advocate of the opinions of that gentleman.

In considering that class of injuries of the abdomen, in which the viscera are not only protruded but injured, Mr. Cooper inquires what method of treatment it is wisest to adopt. He asks whether it is better to return the intestine into the abdomen, and leave the edges of its wound as near as possible to the opening through the parietes, with the hope that nature will secure it there by the adhesive inflammation—or whether it is more advisable for the surgeon to fix it in the place alluded to by means of suture through the bowel or the mesentery? From his own experience, founded on dissection, as well as on experience on the lower animals, he concludes that the following procedure is the best.

"If the wound of the intestine be through the whole of its calibre, extending to the mesentery, or at any rate, implicating a large portion of the cylinder, I then should recommend suture; dangerous as it may seem to be, it is yet to be considered the only means left to secure the position of the intestine, near to the outer wound; but if, on the contrary, when the wound extends only through a small portion of the calibre, the suture need not be used, for nature immediately, by the adhesive inflammation, not only unites the edges of the wound of the intestine and parietes together, but also, by the same process, provides a perfect barrier to the discharge of feculent matter into the cavity of the abdomen." 266.

And again, Mr. Cooper observes—

"I have made several experiments in the endeavour to draw some just inference as to the best mode of treatment in cases of protruding wounds of the intestine, but I fear, from the experiments being made upon lower animals, there is much danger in drawing too hasty deductions from any supposed analogy between the condition in which the human subject would be placed under the same circumstances with them; at any rate, I discovered that the difficulty in preventing the escape of fæces into the abdomen, was exactly in proportion to the size of the opening in the intestine; and would recommend that where the opening was large, whether from accident or disease, as in cases of strangulated hernia, that sutures should be employed in such a manner as to prevent any portion of the wound opening into the cavity of the abdomen. I have never seen an accident of wounded parietes of the abdomen, with protrusion of injured intestine, except in cases of gun-shot wounds, which were removed from my care immediately, and have left no further impression upon my mind, than the recollection of the immediate prostration which follows such accidents. I shall, however, describe two or three cases of strangulated hernia, in which the intestine had been found either sphacelated or ulcerated; and leading therefore to the same surgical considerations as belong to this class of abdominal injury." 267.

*Case.* A man, aged 56, was admitted into Guy's Hospital with a strangulated scrotal hernia, from the symptoms of which he had suffered for nine days. On performing the operation, a large portion of dark-coloured omentum was exposed, covering a considerable knuckle of intestine. This adhered to the sac but was readily separable from it, and although dark coloured, had not, to Mr. Cooper, the appearance of having totally lost its vitality. He accordingly returned it into the abdomen, leaving it as near as possible to the mouth of the sac. Immediately on being put to bed, the patient began to experience a sensation of shivering and cold, whilst the pulse was feeble, and the body bedewed with a cold and clammy perspiration. By means of heat and of stimulants he rallied, but about the middle of the night he was seized with a violent pain in the abdomen, and at five in the morning collapse came on and he died.

Upon examination of the body after death the pelvis was found nearly filled with fæculent matter; and upon searching for the opening in the intestine, through which it had escaped, a small ulcerated orifice was found, accounting at once for the sudden dissolution of the patient. The character of an opening in an intestine, will always shew whether it has been the result of violence or of disease; if of violence, the opening presents a thickened protruded edge, produced by the eversion of the mucous membrane; while on the contrary, if it be ulcerated, the opening presents a thin edge, terminating by peritoneum instead of mucous membrane, in consequence of the much greater rapidity with which ulceration goes on in the mucous, than the serous membranes.

Mr. Cooper has anticipated, but not satisfactorily replied to the objection, that this case is scarcely calculated to illustrate the doctrines previously laid down. The next case is still less appositely introduced, being merely an example of the safety with which portions of omentum may be cut away. He offers an incidental observation on the mode of removing omentum, which appears to be not insusceptible of criticism. He remarks that care must be taken in excising it not to cut through the vessels in which the blood still retains its fluidity, or else there will be occasion for the application of ligatures, which must diminish the hope of recovery from their tendency to produce peritoneal inflammation; and if they be not applied, the danger is equal from the effusion of blood into the cavity of the abdomen. If, indeed, the portion of omentum to which the ligature had been applied were returned into the abdomen, the fear would be founded in reason and experience. Mr. Earle having cut such ligatures close, and returned the whole into the cavity of the peritoneum, abscesses long afterwards formed at the umbilicus, and discharged the knots that had been tied. But Mr. Brodie, and perhaps other surgeons, has advised that the omentum should not be reduced, and that one end of the ligature being left long, should hang at the external wound, and admit of subsequent removal. If it were always possible to comply with Mr. Cooper's proposal, and if we only needed to cut omentum where it would not bleed, we could not contest the superiority of his advice. But we cannot avoid entertaining a doubt, that we cannot at all times practise our incision in such favourable circumstances, and that omental vessels may not bleed when divided, but give rise to hæmorrhage afterwards.

The next point which occupies the attention of our author, is rupture or laceration of the viscera, without any wound in the abdominal parietes. Some interesting cases are presented to our notice. We will try to select and abridge the details.

Case 1.—*Rupture of the Jejunum—Peritonitis.*

A man, æt. 30, was taken to Guy's Hospital Nov. 3d, a lightly laden cart having passed over his abdomen. In the evening Mr. Cooper found him lying on his right side, with his knees drawn up, and his head and shoulders inclined forwards; he complained of violent pain attacking him in paroxysms, like those of colic, attended with great tenderness diffused over the whole abdomen. His abdominal muscles were strongly contracted and tense; his expiration was accompanied by a deep sigh; he had twice vomited, and upon one occasion, a small quantity of blood; his pulse was not accelerated nor feeble, and his skin perfectly warm: leeches were applied to the abdomen, fomentations were also ordered, and thirty drops of tincture of opium were given. A dose of calomel and opium was exhibited at night.

The nausea and other symptoms continued next day, the pulse was

84 and hard, and the tongue was covered with a slight brown fur. *Leeches—calomel and opium—castor oil injection.* Towards noon the pain in the abdomen increased, but the vomiting soon afterwards subsided. *Bowels opened by castor oil injections—an opiate afterwards given.*

After the motion, which contained a small quantity of blood, he immediately complained of a great increase of pain in the right hypochondriac region, the pain being also aggravated by inspiration. The respiration was thoracic—the countenance anxious—decubitus dorsalis—pulse 126, very small and compressible.

He passed a very anxious night—next morning he was sinking, and in the evening he died.

The examination of his body presented the following facts. Externally there was only a slight abrasion to be perceived near the left anterior and superior spinous process of the ilium; no ecchymosis, except from leech-bites, nor any lesion of the abdominal parietes; on opening the cavity of the abdomen, a large quantity of fluid, partly fæculent, but principally serous, was discovered; the peritoneum was extensively inflamed, most intensely in the left iliac region and over the bladder: in both of these situations the intestines were glued together. Upon minute examination of the intestines a laceration was found in the jejunum, which extended transversely through the bowel into the mesentery; the edges of the divided intestine were separated from each other, and the mucous membrane everted. In the cellular membrane, connecting the peritoneum to the lumbar muscles on the left side, a very considerable ecchymosis or infiltration of blood existed, and a similar extravasation was found about the pancreas.

In this case there was no considerable collapse, and the more severe and decisive symptoms followed the purgative effect of an injection. This circumstance is adapted to afford a warning against the rash exhibition of aperients. Perhaps in such a case the abstraction of blood from the arm would be more efficient, in the commencement of the treatment, than the mere application of leeches to the abdomen. We certainly prefer this bolder practice in cases of injury of the three great cavities.

CASE 2.—*Rupture of the Jejunum from a kick upon the Scrotum.*

A man, aged 40, received, at 11 o'clock at night, a kick on the right side of the scrotum. He had never previously observed any hernia, nor indeed any swelling whatever at the part. Immediately after the reception of the blow, he was seized with acute pain in the direction of the cord, succeeded by every symptom of collapse.

Next morning he was admitted into Guy's Hospital, and now he was the subject of an oblique inguinal hernia. This was returned with facility by the dresser, but on moving the patient immediately afterwards, it again came down, and the pain produced by the slightest pressure forbad any further attempt at its reduction. The pulse soon rose to 100, the respiration was hurried, the countenance indicative of suffering, and the pain was chiefly referred to the scrotum.

The progress of the symptoms demands no particular remark. Sickness was established, and the usual features of abdominal inflammation were present. The treatment consisted of three successive leechings—a bleeding to eight ounces, at 5, p.m. of the day of his admission—at 9, p.m. a blister to the abdomen—a dose or two of calomel and opium—and two or three injections. *Dissection.* On laying open the cavity of the abdomen the intestines were found to be highly inflamed, and agglutinated at every point by the effusion of lymph. Upon throwing air into the calibre of the intestines, by means of a blow-pipe, a rupture of the jejunum was discovered about an inch and a half above the internal ring, and through this opening a quantity of fæcal matter had extravasated and inflamed the entire peritoneal cavity, even upon the under surface of the diaphragm. The hernial sac was thickened and ecchymosed at its lower part, and contained a portion of intestine more congested than the rest of the canal.

Mr. Cooper doubts the veracity of the patient, in asserting that he had not been subject to hernia prior to the blow. Perhaps the same remark on the subject of general bleeding might be applicable to this, as to the previous case.

CASE 3.—*Rupture of the Spleen, &c.*

A carter, aged 21, was admitted at 5, p. m. on the 15th October, 1830, a loaded waggon having passed over his loins. The attendants stated that he spat blood on his passage to the hospital. He was received in a condition of extreme collapse. His breathing was frequent, difficult, and rattling, and he expectorated at intervals mucus, tinged with blood. He also complained of great pain in the region of the umbilicus.

At 8, p. m. the pulse was 100, small, feeble, and fluttering—he suffered from extreme restlessness, combined with intolerance of motion in consequence of pain. It had been necessary to draw off the urine with a catheter; six or eight ounces, tinged with blood, were abstracted. *Opiates, with warmth to the feet.*

The symptoms continued, with no further alteration than their mere aggravation would imply. At 6, p. m. of the following day he expired.

*Dissection.* On opening the abdomen, about a pound of blood was discovered, arising from a rupture of the spleen, which was torn into two parts. The diaphragm was also ruptured a little above the œsophageal opening; and there was also an effusion of blood between the liver and peritoneum, in quantity sufficient to separate them to a considerable extent. The kidneys were, in a like manner, separated from their peritoneal covering by blood. The stomach, bladder, and intestines, presented a natural appearance. The inferior part of the left lung was much altered in its appearance, and much gorged with blood. The heart was natural, but there was some effusion between the pericardium and the pleura. A larger quantity of mucus than usual was found in the bronchia. The brain was not examined.

The next and the last case is one of rupture of the kidney. The patient was a boy, and the injury was occasioned by the handle of a truck being swung, with great violence, against his abdomen. The collapse was excessive and immediate, and he died in about an hour and a half after the reception of the injury. On opening the abdomen, a large quantity of coagulated and of fluid blood was discovered in its cavity. This had proceeded from the left kidney, the part of which above the renal vessels was completely severed from that below them.

This concludes our notice of Mr. Cooper's volume. We have not space for even the brief language of ordinary commendation. We would simply recommend it to those who are inclined to study facts.

